# Anti-inflammatory interleukin 1 receptor antagonist concentration in plasma correlates with blood-brain barrier integrity in the primary lesion area in traumatic brain injury patients

**DOI:** 10.1016/j.bbih.2023.100653

**Published:** 2023-06-22

**Authors:** Xuan Vinh To, Patrick Donnelly, Liam Maclachlan, Kate Mahady, Eduardo Miguel Apellaniz, Paul Cumming, Craig Winter, Fatima Nasrallah

**Affiliations:** a Queensland Brain Institute, The University of Queensland, Brisbane, Australia; b Department of Neurosurgery, Royal Brisbane and Women's Hospital, Brisbane, Australia; c School of Health and Rehabilitation Sciences, The University of Queensland, Brisbane, Australia; d Department of Radiology, Royal Brisbane and Women's Hospital, Brisbane, Australia; e Department of Nuclear Medicine, Bern University Hospital, Bern, Switzerland; f School of Psychology and Counselling, Queensland University of Technology, Brisbane, Australia; g Faculty of Medicine, University of Queensland, Brisbane, Australia; h School of Mechanical, Medical and Process Engineering, Queensland University of Technology, Australia

**Keywords:** Traumatic brain injury, Cerebral contusion, Dynamic contrast enhancement, Susceptibility-weighted imaging, Cerebral microbleed, Micro haemorrhage, Blood-brain barrier dysfunction

## Abstract

**Purpose:**

Blood-brain barrier (BBB) dysregulation and pro-inflammatory signalling molecules are secondary factors that have been associated with injury severity and long-term clinical outcome following traumatic brain injury (TBI). However, the association between BBB permeability and inflammation is unknown in human TBI patients. In this study, we investigated whether BBI integrity as measured by Dynamic Contrast-Enhanced (DCE) Magnetic Resonance Imaging (MRI) correlates with plasma levels of immunological markers following TBI.

**Methods:**

Thirty-two TBI patients recruited from a neurosurgical unit were included in the study. Structural three-dimensional T1-weighted and DCE-MRI images were acquired on a 3T MRI at the earliest opportunity once the participant was sufficiently stable after patient admission to hospital. Blood sampling was performed on the same day as the MRI. The location and extents of the haemorrhagic and contusional lesions were identified. Immunological biomarkers were quantified from the participants’ plasma using a multiplex immunoassay. Demographic and clinical information, including age and Glasgow Coma Scale (GCS) were also collected and the immunological biomarker profiles were compared across controls and the TBI severity sub-groups. Contrast agent leakiness through blood-brain barriers (BBB) in the contusional lesions were assessed by fitting DCE-MRI using Patlak model and BBB leakiness characteristics of the participants were correlated with the immunological biomarker profiles.

**Results:**

TBI patients showed reduced plasma levels of interleukin (IL)-1β, IFN-γ, IL-13, and chemokine (C–C motif) ligands (CCL)2 compared to controls and significantly higher levels of platelet-derived growth factor (PDGF-BB), IL-6, and IL-8. BBB leakiness of the contusional lesions did not significantly differ across different TBI severity sub-groups. IL-1ra levels significantly and positively correlated with the contusional lesion's BBB integrity as measured with DCE-MRI via an exponential curve relationship.

**Discussion:**

This is the first study to combine DCE-MRI with plasma markers of inflammation in acute TBI patients. Our finding that plasma levels of the anti-inflammatory cytokine IL-1ra correlated negatively with increased leakiness of the BBB.

## Highlights

• Patterns of both favourable and unfavourable outcomes in blood immunological markers post-Traumatic Brain injury (TBI).
• Degrees of blood-brain barrier (BBB) leakiness in the primary contusions does not vary significantly across TBI severities.
• Higher levels of interleukin 1 receptor antagonist (IL-1ra) were associated with lower levels of BBB leakiness.
• IL-1ra effect on the BBB may be more due baseline variation in individuals’ IL-1ra levels prior to the TBIs.

## 1 Introduction

Traumatic brain injury (TBI) entails structural injury to the brain, which commonly results in persistent neurological, psychosocial, and/or cognitive impairments (Amyot et al., 2015). The severity of the TBI is commonly defined according to the Glasgow Coma Scale (GCS) in the acute phase (Hawryluk and Manley, 2015; Teasdale and Jennett, 1974), but there is great complexity and diversity in the nature and consequences of TBI in the short- and long-terms, which can include neural cell loss, axonal damage and demyelination, disconnection cortical and subcortical structures, and neuroinflammation (Hutchinson et al., 2018; Loane and Byrnes, 2010; Harris et al., 2016; Ramlackhansingh et al., 2011). Neuroinflammation post-TBI represents a complex interaction between central and peripheral signalling molecules, and is influenced by the patient's baseline physiology (age, sex, genetic variation), the TBI itself and its secondary injuries, and effects of treatment (Simon et al., 2017). The initial trauma provokes damage to the blood-brain barrier (BBB) and promotes a neuroinflammatory cascade that includes initial signalling, microglia activation, altered gene expression, complement activation, peripheral immune cell recruitment, involvement of the adaptive immune system, and astrogliosis. This cascade is not one-way or linear process, but rather involves feedback between reciprocal pro-inflammatory and anti-inflammatory pathways and mechanisms (Zheng et al., 2022). Neuroinflammation is neither entirely detrimental nor beneficial *per se*, but engages detrimental pathways promoting neuronal death and neurodegeneration, as well as restorative mechanisms that promote clearance of cellular debris and neuronal regeneration (Zheng et al., 2022). Minimally invasive assessment of neuroinflammation relies on an immunological marker panel or profile from peripheral blood, which has been gaining attention as a prognostic indicator, while also presenting new interventional targets for improving a patient's outcome post-TBI (Needham et al., 2019). Positron emission tomography (PET) using radiolabelled ligands selective for the 18 kDa translocator protein (TSPO) remains one of the most widely adopted imaging technique to assess neuroinflammation *in vivo*, though it is also known that changes TSPO levels can be of non-inflammatory process (Notter et al., 2018).

Most notably, plasma and cerebrospinal fluid levels of tumour necrosis factor alpha (TNF-***α***) and various interleukins (IL-1β, IL-6, IL-8, and IL-10) have been indicated as markers for severity classification and prognosis for TBI (Woodcock and Morganti-Kossmann, 2013). Among the other blood markers that have also been considered are the S100 astroglial calcium-binding protein beta (S100β), glial fibrillary acidic protein (GFAP), neuronal specific enolase (NSE), and ubiquitin C-terminal hydrolase-L1 (UCH-L1) (Zetterberg and Blennow, 2016; Vos et al., 2004; Chabok et al., 2012; Mercier et al., 2013). Nevertheless, earlier studies of inflammatory biomarkers in the TBI context have often adopted a reductionist approach focusing on a single biomarker, thus oversimplifying the complex interactions among the various cytokines and chemokines, and other markers of cellular damage.

The integrity of the BBB is often compromised following TBI (Alluri et al., 2015), which can exacerbate disturbances in the delicate homeostasis required to regulate immunological reactions in the nervous system (Zheng et al., 2022). Post-traumatic activation of glial cells and the neuroinflammatory cascade and lead to further BBB dysfunction, and infiltration of immune cells through the compromised BBB, which in turn can further aggravate glial cell activation and inflammation (Takata et al., 2021). While immune cells passage into the brain is rare in the absence of neuroinflammation, T cells can still infiltrate the brain through an intact BBB (Engelhardt et al., 2017); though the crossing of activated T cells leads to release of proinflammatory cytokines that changes the BBB characteristics and recruits additional immune cells through the BBB (Engelhardt et al., 2017). A such, BBB dysfunction and neuroinflammation can initiate a positive-feedback progression of neuropathology long after the initial insult (Takata et al., 2021). Dynamic Contrast-Enhanced MRI (DCE-MRI) is an MRI modality with serial acquisition of T1-weighted images before, during, and after intravascular injection of a contrast agent. The degree and extent of BBB dysfunction can thereby be quantified from the dynamic accumulation of a Gadolinium-based contrast agent (GBCA) in the extravascular-extracellular space, from which the volume transfer rate (K^trans^) is computed (Armitage et al., 2011).

To date, there is no investigation of plasma inflammation markers in relation to BBB dysfunction in sub-acute TBI patients. Given the cascade of biological events that follows a TBI, we tested the hypothesis that levels of plasma markers of inflammation in the aftermath of a TBI would correlate with the extent of BBB dysfunction measured by DCE-MRI. We further predicted that plasma markers of inflammation, i.e., cytokines and chemokines, would bear a relation to severity of injury and clinical outcome.

## 2 Materials and methods

### 2.1 Study subjects and design

In this prospective study approved by the institutional Human Research Ethics Committee (approval number HREC/16/QRBW/604), patients were approached after their admission to the participating hospital's neurosurgical unit following a TBI event. The participating hospital is a level-1 trauma hospital serving the Metro North Health network (Brisbane, Queensland, Australia) with 900,000 population. We recruited patients between June 2009 and October 2021, with clustering around two time-periods: P01 to P15 were recruited in 2009–2010 and P16 to P32 were recruited in 2018–2022. We obtained written, informed consent from either the patient or a legally sanctioned guardian. Exclusion criteria were age under 18 or over 80 years, a history of neurodegenerative diseases or significant pre-existing mental health disorders, or if the MRI was contraindicated *i.e.,* known allergy to the contrast agent, or presence of a pacemaker. No patient was no immunosuppressant or anti-inflammatory medication, and the use of aspirin was withheld prior to patients went on the study.

All patients were indicated for CT at admission, and proved to have significant findings on the initial clinical CT, or met the National Institute of Neurological Disorders and Stroke's (NINDS) (Haacke et al., 2010) or American Congress of Rehabilitation Medicine's (ACRM) (Kay et al., 1993) definition of mild TBI, namely a documented loss of consciousness following a head injury, or a period of post-traumatic amnesia. Some data obtained from 20 out of 32 subjects in this study (P01 – P20) were reported in a prior study (Nichols et al., 2021).

We recruited a group of 19 demographically matched healthy controls for donation of blood samples. Selection criteria for the controls were absence of significant neurodegenerative or mental health disorders, and no diagnosed TBI within the preceding 12 months. The control group did not undergo MRI scans.

### 2.2 MRI acquisition

#### 2.2.1 Structural imaging

We aimed to scan the TBI participants at the earliest scanner availability after they were determined by the treating physician to be clinically stable. The imaging time-point post-TBI was (median/interquartile range) 4 days/1–9 days post-TBI. For most participants, the imaging time-point reflected the time required to become sufficiently stable post-TBI, although P28 and P29 were imaged 213 and 114 days post-TBI, respectively, due to COVID-19-related restrictions and scanner availability. The MRI scans were conducted on a 3-T MRI scanner (Prisma, Siemens Healthcare, Germany) using a 32-channel head array coil. Anatomical T1-weighted (T1w) Magnetisation Prepared Rapid Gradient-Echo (MPRAGE) scan, with the following parameters for participants P01 to P15: TE = 2.4 ms, TR = 900 ms, FA = 9 degrees, effective resolution = 1 mm^3^ isotropic. Participants P16 to P32 were imaged with the following parameters: TE = 2.26 ms, TR = 1900 ms, FA = 9 degrees, effective resolution = 1 mm^3^ isotropic. The structural T1-weighted Magnetisation Prepared Rapid Gradient-Echo (T1w-MPRAGE) sequence was performed before and after to the injection of Gadolinium-based contrast agent (GBCA). Anatomical T2-weighted (T2w)-Fluid-Attenuated Inversion Recovery (FLAIR) imaging was performed before the administration of GBCA. We performed the T2w FLAIR imaging with the following parameters for participants P01 to P15: TE = 87 ms, TR = 9000 ms, FA = 150 degrees, and effective resolution = 0.9 × 0.9 × 7.5 mm. For participants P16 to P32, the T2w FLAIR images were acquired as follows: TE = 81 ms, TR = 9000 ms, FA = 150 degrees, and effective resolution = 0.69 × 0.69 × 3.3 mm.

#### 2.2.2 Dynamic contrast enhancement Magnetic Resonance Imaging (DCE-MRI) acquisition

DCE-MRI acquisitions consisted of a series of 3D gradient-echo (GRE) T1-weighted Controlled Aliasing in Parallel Imaging Results in Higher Acceleration-Volumetric Interpolated Breath-hold Examination sequence (CAIPIRINHA-VIBE) volumes. We scanned participants P01 to P15 with the following parameters: TE = 3 ms, TR = 8.1 ms, FA = 12 degrees, effective resolution = 0.94 × 0.94 × 4 mm, temporal resolution = 55 s, and acquisition of 26 volumes. We scanned participants P16 to P32 with the following parameters: TE = 1.8 ms, TR = 4.35 ms, FA = 9 degrees, effective resolution = 0.94 × 0.94 × 0.9 mm, temporal resolution = 66 s, and acquisition of 20 volumes. We obtained baseline volumes for the first 2 min, with bolus application to an antecubital vein of 0.1 mmol/kg Gadovist (GE Healthcare, USA) contrast agent while the dynamic acquisition was running.

### 2.3 MRI image processing

#### 2.3.1 Structural image processing

Brain extraction/skull stripping was performed on pre- and post-GBCA structural T1w MPRAGE images using FSL BET (Smith, 2002). Next, the brain-extracted pre- and post-GBCA T1w MPRAGE images of each participant were rigidly registered together using ANTS (v.2.3.4) (Avants et al., 2009). The non-skull-stripped T2w FLAIR and pre-GBCA T1w MPRAGE images of each participant were also rigidly registered together. The magnitude images of the first two echoes of the SWI acquisition were averaged together to create a spatial reference image of the SWI sequence. This spatial reference image was skull-stripped using FSL BET, and the skull-stripped reference image was rigidly registered to the skull-stripped pre-GBCA T1w MPRAGE, whereupon the susceptibility-weighted image was transformed into the pre-GBCA T1w MPRAGE space.

Registered pre- and post-GBCA T1w MPRAGE images were subtracted from each other to create a GBCA-enhancement map. The Haemorrhagic Contusion Core (HCC) consists of the haemorrhagic, contused brain parenchyma and vasogenic oedema lesion, and is defined as the volume that is hyper-intense on T2w FLAIR, hypointense or isointense with normal grey matter on T1w MPRAGE, and with elevated GBCA enhancement (Nichols et al., 2021). The HCC mask was semi-automatically segmented using region competition snakes segmentation (Ho et al., 2003) available in ITK-SNAP (v.3.8) with the multi-modal tissue class classifier pre-segmentation step used to generate the preliminary segmentation probabilistic map. In brief, the co-registered pre-GBCA T1w MPRAGE image, T2w FLAIR image, and GBCA-enhancement map of each participant were loaded into ITK-SNAP. Representative regions of the HCC (as defined above), normal grey matter, normal white matter, and the cerebrospinal fluid (CSF; hypointense on both T1w MPRAGE and T2w FLAIR) were identified manually for each participant's data and used as tissue class classifiers for the semi-automatic classification pre-segmentation. The pre-segmentation tissue class probabilistic maps were inspected (by XVT) to ensure that they reasonably delineated the four different tissue classes. Next, we seeded initiation bubbles on suspected HCC regions of the pre-segmented probabilistic map, and then use the bubbles to fill out the HCC mask by iterative snake evolution; here, the curvature parameter was set to 0.001.

Pre-GBCA T1w MPRAGE images were segmented into grey matter, white matter, and CSF probabilistic maps using SPM12's Segment tool. The white matter and grey matter tissue probabilistic maps were thresholded at >95% probability, binarized, combined, and subtracted by the HCC masks to create a normal-appearing brain (NAB) mask. The NAB mask represents grey and white matter areas devoid of obvious lesions, haemorrhages, or microbleeds.

#### 2.3.2 DCE-MRI processing

DCE-MRI were processed similarly as described in an earlier manuscript (Nichols et al., 2021). In brief, the image data were motion-corrected to the first baseline volume using FSL's mcflirt (Smith et al., 2004), followed by perfusion model fitting using the ROCKETSHIP toolbox (Barnes et al., 2015). A vascular image derived input function (IDIF) was extracted from a 1 mm^3^ isotropic region-of-interest (ROI) place in the lumen of the middle cerebral artery bilaterally and in the superior sagittal sinus of the motion-corrected DCE-MRI volume. Since no acquired T1 map was available for this study, we generated a surrogated T1 map by assigning a homogenous T1 value of the grey matter, white matter, and CSF (Wright et al., 2008) to all of the voxels of each of the respective grey matter, white matter, and CSF masks from the T1w MPRAGE segmentation step. Thus, the model fitting of the DCE-MRI data was relative and semi-quantitative. Semi-quantitative BBB permeability maps (K^trans^; min^−1^) were calculated using the IDIF by Patlak linear graphic analysis (Patlak and Blasberg, 1985). We quantified average K^trans^ values of the HCC, NAB, and the ratio of HCC/NAB K^trans^ ratio from the individual K^trans^ maps upon applying the relevant masks. If a participant had no identifiable HCC, the HCC/NAB K^trans^ ratio was set to unity.

### 2.4 Blood sampling, processing, and quantification of immune cytokine concentration

On the same day of the MRI examination, we collected blood samples in EDTA collection tubes for the TBI participants and centrifuged 10 ml samples at 1600 g for 10 min, with removal of the plasma fraction for storage at −80 °C until analysis. We then conducted a multiplexed immunoassay using the Bio-Plex Pro Human Cytokine 27-plex Assay (Catalogue number: M500KCAF0Y) following the manufacturer's instructions without modification. In brief, the frozen samples were thawed, vortexed, and centrifuged at 1000 g for 2 min at 4 °C. As per the manufacturer's instructions, samples were diluted 1:4 with the sample diluent from the kit and then passed through 0.22 μm high recovery Durapore (PVDF) centrifuge filters at 14,000 g for 15 min at 4 °C. All standards and samples were analysed on a 96-well plate with a robotic liquid handling workstation (epMotion 5075; Eppendorf). The plate was washed on a Bio-Plex Pro II magnetic plate washer (Bio-Rad) and read with the Bio- Plex Systems 200. During incubation at 25 °C, the assay plates were shaken at 850 rpm in subdued light. Samples were analysed and standard curves generated using the Bio-Plex Manager v6.0 software. For analysis and quantification, if the observed concentration of an analyte fell below the detectable range of the standard curves, we set that value to zero. The following plasma inflammatory marker levels were quantified: interleukin IL-1β, IL-4, IL-6, IL-7, IL-8, IL-9, IL-17, interleukin 1 receptor antagonist (IL-1ra), Eotaxin, basic fibroblast growth factor (bFGF), granulocyte colony-stimulating factor (G-CSF), interferon gamma (IFN-γ), chemokine (C–C motif) ligands 2, 3, 4, and 5 (CCL2, CCL3, CCL4, and CCL5), C-X-C motif chemokine ligand 10 (CXCL10), platelet-derived growth factor-BB (PDGF-BB), and tumour necrosis factor alpha (TNF-***α***).

### 2.5 Statistical analysis

Statistical analysis was performed in GraphPad Prism (v.9.3.1, GraphPad Software, Inc., San Diego, California, USA). Normality of the data was tested using the Shapiro-Wilk test (Shapiro and Wilk, 1965; Yap and Sim, 2011). TBI participants were assigned to three sub-groups according to TBI severity as classified using the Glasgow Coma Scale (GCS) (Hawryluk and Manley, 2015; Teasdale and Jennett, 1974), as mild (GCS = 13–15), moderate (GCS = 9–12), and severe (GCS <9). Comparison of imaging metrics quantified from MRIs, HCC K^trans^, NAB K^trans^, and HCC/NAB K^trans^ ratio across TBI sub-groups were performed using the Kruskal-Wallis one-way Analysis of Variance (ANOVA) test (Kruskal and Wallis, 1952). Comparisons of plasma inflammatory marker levels were performed similarly, but with pair-wise *post-hoc* tests comparing each of the TBI sub-groups and the control group, and with correction for multiple comparisons using the two-stage linear step-up procedure of Benjamini, Krieger, and Yekutieli false discovery rate correction (Benjamini et al., 2006). Since the TBI sub-groups were imbalanced, with relatively more mild TBIs compared to moderate-severe, a separate Mann-Whitney test comparing the ranks of cytokine levels between all TBI participants and all controls were performed and the results were also corrected for multiple comparisons using false discovery rate correction.

Relationships between each of the imaging metrics (HCC K^trans^, NAB K^trans^, and HCC/NAB K^trans^ ratio) and each of the plasma inflammatory marker's level were analysed using Spearman's non-parametric correlation analysis; normality test indicated that the imaging metrics and plasma inflammatory markers' levels were non-parametric, and the relationship were non-linear. For each of the imaging metric and plasma inflammatory marker level pair that was shown to be significantly correlating, the relationship is further examined by comparing linear, mono-exponential decay, and mono-exponential growth function models with selection of the better model by Akaike's Information Criterion (Akaike et al., 1998). Next, we fitted the higher likelihood model to the data by least squares fitting. If the slope was significantly different from zero, we applied the Shapiro-Wilk normality test on the distribution of the residuals of the linear regression, and similarly for the residuals for cases with greater likelihood of a mono-exponential function. The linear model was significant if the slope differed from zero and the residuals passed the normality test, whereas the mono-exponential growth or decay function was significant if the residuals passed the normality test.

The tests were thresholded and considered significant at P value < 0.05, or Q value < 0.05 in case of the two-stage linear step-up procedure of Benjamini, Krieger, and Yekutieli false discovery rate correction.

## 3 Results

### 3.1 Inflammatory marker profiles of TBI participants vs. controls

**Fig. 1:**
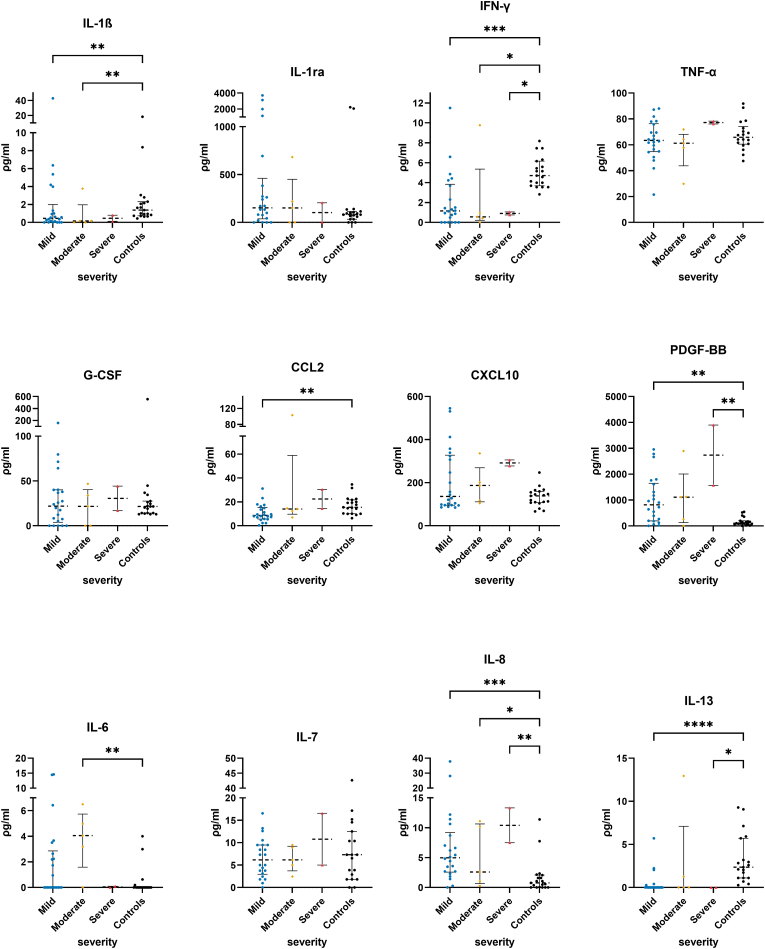
Quantified plasma concentrations of immunological biomarkers from traumatic brain injury severity sub-groups and control group of interleukin (IL)-1β, interleukin 1 receptor antagonist (IL-1ra), interferon gamma (IFN-γ), tumour necrosis factor alpha (TNF-***α***), granulocyte colony-stimulating factor (G-CSF), chemokine (C–C motif) ligands 2 (CCL2), C-X-C motif chemokine ligand 10 (CXCL10), platelet-derived growth factor-BB (PDGF-BB), IL-6, IL-7, IL-8, IL-13. Pair-wise *post-hoc* tests comparing each of the TBI sub-groups and the control group, and with correction for multiple comparisons using the two-stage linear step-up procedure of Benjamini, Krieger, and Yekutieli false discovery rate correction, *Q value < 0.05, **Q value < 0.01, ***Q value < 0.001, ****Q value < 0.0001. Bars and whiskers presented the median and inter-quartile range.

**Fig. 2:**
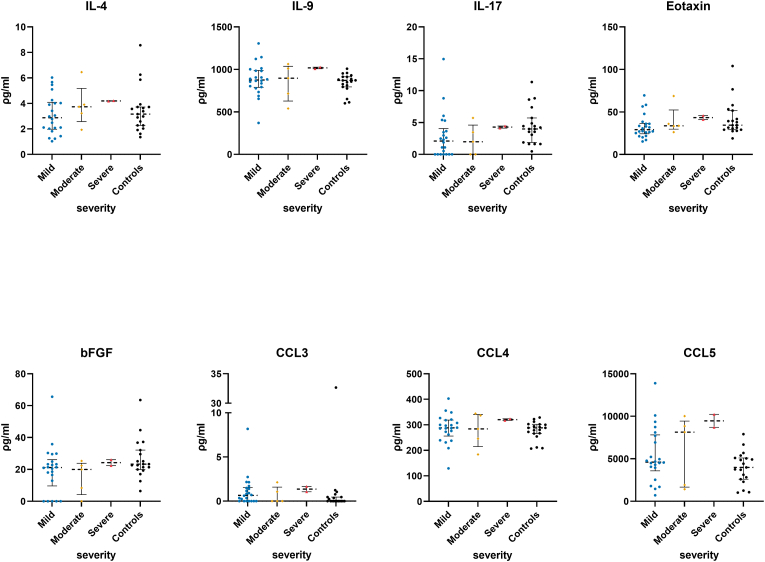
Quantified plasma concentrations of immunological biomarkers from traumatic brain injury severity sub-groups and control group of interleukin (IL)-1β, interleukin (IL)-4, IL-9, IL-17, Eotaxin, basic fibroblast growth factor (bFGF), chemokine (C–C motif) ligands 2 (CCL3), CCL4, and CCL5. Pair-wise *post-hoc* tests comparing each of the TBI sub-groups and the control group, and with correction for multiple comparisons using the two-stage linear step-up procedure of Benjamini, Krieger, and Yekutieli false discovery rate correction. No significant found. Bars and whiskers presented the median and inter-quartile range.

Shapiro-Wilk's normality test indicated that participants' age, initial GCS, imaging outcomes (HCC K^trans^, NAB K^trans^, and HCC/NAB K^trans^ ratio) and most inflammation markers (except IL-4, IL-7, IL-9, PDGF-BB, CCL4, CCL5, and TNF-α) did not follow a Gaussian distribution (Table 1). Kruskal-Wallis one-way ANOVA test and *post-hoc* tests showed TBI participants had significantly different plasma levels of several markers compared to the healthy controls (Fig. 1). TBI participants had significantly lower plasma levels of IL-1β (mild vs. controls: Q = 0.008, moderate vs. control; Q = 0.008), IFN-γ (mild vs. controls: Q = 0.0008, moderate vs. controls: Q = 0.017, severe vs. controls: Q = 0.047), IL-13 (mild vs. control: Q < 0.0001, severe vs. controls: Q = 0.0222) and CCL2 (mild vs. controls: Q = 0.0071). TBI participants had significantly higher plasma levels of PDGF-BB (mild vs. controls: Q = 0.003 and severe vs. controls, Q = 0.0093), IL-6 (moderate vs. controls, Q = 0.0078) and IL-8 (mild vs. controls: Q = 0.0003, moderate vs. control: Q = 0.0415, and severe vs. controls: Q = 0.0057). The remaining markers showed no significant differences between TBI sub-groups and controls (Fig. 1 – Il-1ra, TNF-***α***, G-CSF, CSCL10, IL-7 and Fig. 2).

**Table 1:** Shapiro-Wilk's normality test results and the median and inter-quartile range of the different variables quantified from the study's participants.

	GCS	Age	HCC Ktrans	NAB ktrans	HCC/NAB ratio	IL-1β (ρg/mL)	IL-1ra (ρg/mL)	IL-4 (ρg/mL)	IL-6 (ρg/mL)	IL-7 (ρg/mL)	IL-8 (ρg/mL)	IL-9 (ρg/mL)	IL-13 (ρg/mL)	IL-17 (ρg/mL)	Eotaxin (ρg/mL)	bFGF (ρg/mL)	G-CSF (ρg/mL)	IFN-γ (ρg/mL)	CXCL10 (ρg/mL)	CCL2 (ρg/mL)	CCL3 (ρg/mL)	PDGF-BB (ρg/mL)	CCL4 (ρg/mL)	CCL5 (ρg/mL)	TNF-α (ρg/mL)
Shapiro-Wilk test																									
W	0.7823	0.943	0.8641	0.8342	0.7764	0.328	0.56	0.9566	0.6697	0.9503	0.7288	0.9728	0.3622	0.7981	0.8959	0.8584	0.7782	0.7109	0.849	0.5335	0.6324	0.8889	0.9615	0.9445	0.9498
P value	<0.0001	0.1202	0.0015	0.0004	<0.0001	<0.0001	<0.0001	0.2705	<0.0001	0.1864	<0.0001	0.6376	<0.0001	<0.0001	0.0078	0.0011	<0.0001	<0.0001	0.0007	<0.0001	<0.0001	0.0054	0.3569	0.1312	0.1811
Passed normality test (alpha = 0.05)?	No	Yes	No	No	No	No	No	Yes	No	Yes	No	Yes	No	No	No	No	No	No	No	No	No	No	Yes	Yes	Yes

25% Percentile	12.5	24	0.007223	0.00085	3.78	0	10.36	2.05	0	3.44	2.44	785.8	0	0	26.12	10.63	2.6	0.215	100.5	7.06	0	228.2	253.7	3233	55.75
Median	14	41	0.0185	0.0016	13.8	0.35	151.7	3.23	0.1	6.17	5.1	889.2	0	2.31	33.05	21.35	21.72	0.94	157.9	9.61	0.7	961.4	289	4770	64.18
75% Percentile	15	54	0.0408	0.002554	31.73	1.16	327.6	4.195	3.58	9.49	10.4	1009	0	4.29	39.84	25.12	40.14	2.995	315.4	15.5	1.58	1685	322.1	8710	75.95

Mann-Whitney test comparing the cytokine levels, corrected for multiple comparisons between TBI participants and controls are presented in Table 2. IL-1β, IL-13, IL-17, IFN-γ, and CCL2 were lower in TBIs than controls while IL-6, IL-8, and PDGF-BB were higher in TBIs than controls.

**Table 2:** Mann-Whitney test results comparing the quantified plasma immunological markers between all Traumatic Brain Injury participants and all the controls.

	IL-1β (ρg/mL)	IL-1ra (ρg/mL)	IL-4 (ρg/mL)	IL-6 (ρg/mL)	IL-7 (ρg/mL)	IL-8 (ρg/mL)	IL-9 (ρg/mL)	IL-13 (ρg/mL)	IL-17 (ρg/mL)	Eotaxin (ρg/mL)	bFGF (ρg/mL)	G-CSF (ρg/mL)	IFN-γ (ρg/mL)	CXCL10 (ρg/mL)	CCL2 (ρg/mL)	CCL3 (ρg/mL)	PDGF-BB (ρg/mL)	CCL4 (ρg/mL)	CCL5 (ρg/mL)	TNF-α (ρg/mL)
Mann-Whitney test																				
P value	**0.0027**	0.1964	0.921	**0.0194**	0.8713	**<0.0001**	0.2759	**<0.0001**	**0.0243**	0.1455	0.0769	0.7818	**<0.0001**	0.147	**0.0132**	0.0474	**0.0001**	0.4146	0.0878	0.4674
FDR correction (Q < 0.05) Significant difference TBIs vs. controls	**Yes**	No	No	**Yes**	No	**Yes**	No	**Yes**	**Yes**	No	No	No	**Yes**	No	**Yes**	No	**Yes**	No	No	No
Median of controls	1.35	81.21	3.16	0.00	7.33	0.76	870.20	2.35	3.99	34.59	23.28	21.60	4.71	139.00	15.48	0.70	101.10	288.00	3979.00	67.51
Median of TBIs	0.35	151.70	3.23	0.10	6.17	5.10	889.20	0.00	2.31	33.05	21.35	21.72	0.94	157.90	9.61	0.70	961.40	289.00	4770.00	64.18

### 3.2 Quantified imaging metrics of DCE

**Fig. 3:**
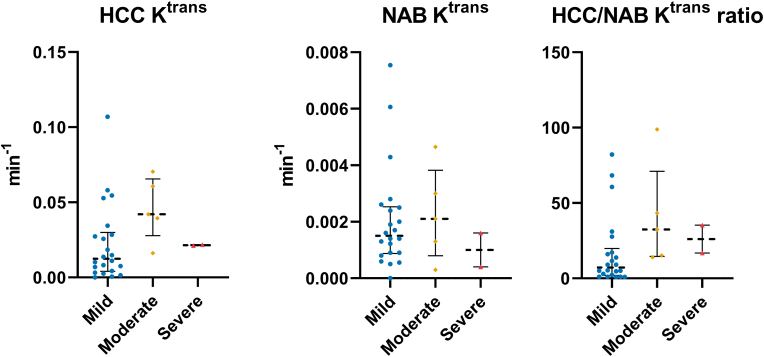
Transfer constant (K^trans^) Dynamic Contrast Enhanced Magnetic Resonance imaging quantified from the Haemorrhagic Contusion Core (HCC) and the Normal Appearing Brain (NAB) area and the ratio of HCC and NAB K^trans^. Kruskal-Wallis one-way Analysis of Variance (ANOVA) test. No significant difference. Bars and whiskers presented the median and inter-quartile range.

The Kruskal-Wallis one-way ANOVA test showed no significant differences across the mild, moderate, and severe TBI sub-groups in the quantified imaging metrics: HCC K^trans^, NAB K^trans^, or HCC/NAB K^trans^ ratio (Fig. 3 and Table 3).

**Table 3:** Kruskal-Wallis one-way Analysis of Variance (ANOVA) test and air-wise *post-hoc* tests comparing each of the TBI sub-groups and the control group with multiple comparison correction using the two-stage linear step-up procedure of Benjamini, Krieger, and Yekutieli false discovery rate correction, thresholded at P value < 0.05 or Q value < 0.05, of the transfer constant (K^trans^) from the Haemorrhagic Contusion Core (HCC), Normal Appearing Brain (NAB), and the ratio of K^trans^ of the HCC and NAB.

(a) HCC Ktrans		
**Kruskal-Wallis one-way ANOVA test**	P value =	0.068
**Post-hoc test (FDR-corrected)**	Q value	
Mild vs. Moderate	0.067	
Mild vs. Severe	0.617	
Moderate vs. Severe	0.593	

(b) NAB Ktrans		
**Kruskal-Wallis one-way ANOVA test**	P value =	0.544
**Post-hoc test (FDR-corrected)**	Q value	
Mild vs. Moderate	0.657	
Mild vs. Severe	0.562	
Moderate vs. Severe	0.562	

(c) HCC/NAB Ktrans ratio		
**Kruskal-Wallis one-way ANOVA test**	P value =	0.050
**Post-hoc test (FDR-corrected)**	Q value	
Mild vs. Moderate	0.093	
Mild vs. Severe	0.263	
Moderate vs. Severe	0.991	

### 3.3 Relationship between plasma levels of immunological markers and imaging outcomes

**Fig. 4:**
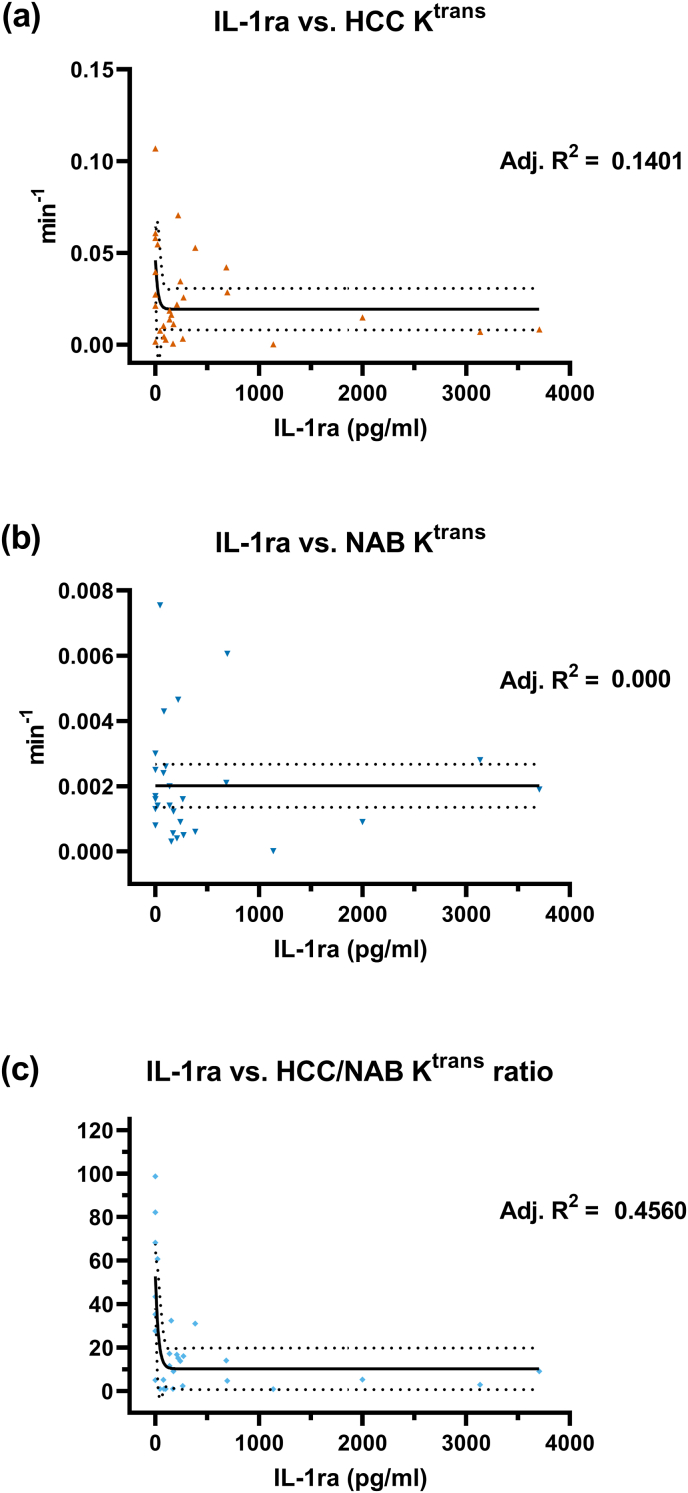
Fitting of the relationship between plasma interleukin 1 receptor antagonist (IL-1ra) levels and the transfer constant (K^trans^) Dynamic Contrast Enhanced Magnetic Resonance imaging quantified from the (a) Haemorrhagic Contusion Core (HCC) and (b) the Normal Appearing Brain (NAB) area and (c) the ratio of HCC and NAB K^trans^. (a) and (c): mono-exponential decay relationship was more likely. (b) linear relationship was more likely.

Correlation model fitting analysis showed that plasma levels of IL-1β, IFN-γ and IL-1ra had negative correlation with HCC K^trans^, and HCC/NAB K^trans^ ratio (Table 4). Model fitting found only the plasma levels of IL-1ra to have a suitable linear or exponential relationship with any of the imaging metric. Plasma IL-1ra level followed a mono-exponential decay relationship (adjusted R^2^ = 0.14) with HCC K^trans^ (Fig. 4a), with the distribution of the residuals passing the normality test (Shapiro Wilk test, P value = 0.052). The empirical equation for the relationship between HCC K^trans^ (Y, min^−1^) and IL-1ra plasma concentration (X, pg/ml) was as follows:

$$Y=\left(0.04594-0.01928\right)\times e^{-0.0427X}+0.01928$$

**Table 4:** 95% confidence intervals of Spearman correlation coefficient of the quantified plasma immunological markers and initial injury severity, Glasgow Coma Scale, Age, and the transfer constant (K^trans^) from the Haemorrhagic Contusion Core (HCC), Normal Appearing Brain (NAB), and the ratio of K^trans^ of the HCC and NAB.

	severity	GCS	Age	HCC Ktrans	NAB ktrans	HCC/NAB ratio
severity		−0.887 to −0.549	−0.604 to 0.0914	**0.00505 to 0.662**	−0.406 to 0.345	**0.0978 to 0.711**
GCS	−0.887 to −0.549		−0.000583 to 0.659	−0.589 to 0.115	−0.255 to 0.486	**−0.671 to -0.0217**
Age	−0.604 to 0.0914	−0.000583 to 0.659		−0.467 to 0.277	−0.291 to 0.456	−0.634 to 0.0428
HCC Ktrans	**0.00505 to 0.662**	−0.589 to 0.115	−0.467 to 0.277		−0.428 to 0.323	0.616 to 0.907
NAB ktrans	−0.406 to 0.345	−0.255 to 0.486	−0.291 to 0.456	−0.428 to 0.323		−0.575 to 0.136
HCC/NAB ratio	**0.0978 to 0.711**	**−0.671 to -0.0217**	−0.634 to 0.0428	0.616 to 0.907	−0.575 to 0.136	
IL-1β	−0.512 to 0.223	−0.335 to 0.417	**0.0164 to 0.668**	**−0.662 to -0.00530**	−0.132 to 0.578	**−0.744 to -0.167**
IL-1ra	−0.514 to 0.219	−0.358 to 0.394	−0.148 to 0.566	−0.564 to 0.152	−0.501 to 0.236	**−0.712 to -0.0995**
IL-4	−0.127 to 0.581	−0.623 to 0.0622	−0.0296 to 0.642	−0.322 to 0.428	−0.600 to 0.0979	−0.311 to 0.438
IL-6	−0.201 to 0.528	−0.719 to −0.113	−0.467 to 0.277	−0.379 to 0.374	−0.325 to 0.425	−0.271 to 0.473
IL-7	−0.261 to 0.481	−0.513 to 0.221	−0.0565 to 0.626	−0.478 to 0.265	−0.586 to 0.119	−0.287 to 0.459
IL-8	−0.326 to 0.424	−0.586 to 0.119	−0.495 to 0.243	−0.475 to 0.268	−0.495 to 0.244	−0.386 to 0.366
IL-9	−0.226 to 0.509	−0.575 to 0.136	−0.374 to 0.379	−0.421 to 0.330	−0.544 to 0.180	−0.344 to 0.408
IL-13	−0.435 to 0.315	−0.171 to 0.550	−0.00460 to 0.657	−0.611 to 0.0809	−0.0102 to 0.653	−0.708 to −0.0908
IL-17	−0.268 to 0.476	−0.486 to 0.255	0.0218 to 0.671	−0.533 to 0.195	−0.470 to 0.274	−0.514 to 0.220
Eotaxin	−0.0645 to 0.621	**−0.701 to -0.0784**	−0.275 to 0.470	−0.0486 to 0.631	−0.638 to 0.0371	−0.102 to 0.598
bFGF	−0.357 to 0.395	−0.465 to 0.280	−0.256 to 0.485	−0.613 to 0.0777	−0.579 to 0.130	−0.521 to 0.210
G-CSF	−0.380 to 0.373	−0.451 to 0.297	−0.330 to 0.421	−0.649 to 0.0181	−0.571 to 0.141	−0.563 to 0.154
IFN-γ	−0.477 to 0.266	−0.222 to 0.512	**0.00229 to 0.661**	**−0.720 to -0.116**	−0.0726 to 0.616	**−0.829 to -0.375**
CXCL10	−0.240 to 0.498	−0.549 to 0.173	**−0.702 to -0.0804**	−0.0261 to 0.644	−0.494 to 0.245	−0.0382 to 0.637
CCL2	−0.0296 to 0.642	−0.598 to 0.101	−0.122 to 0.584	−0.452 to 0.295	−0.506 to 0.230	−0.448 to 0.300
CCL3	−0.380 to 0.372	−0.469 to 0.275	−0.574 to 0.137	−0.431 to 0.319	−0.657 to 0.00331	−0.272 to 0.472
PDGF-BB	−0.198 to 0.531	**−0.685 to -0.0466**	−0.589 to 0.115	−0.444 to 0.305	−0.569 to 0.144	−0.206 to 0.525
CCL4	−0.278 to 0.467	−0.498 to 0.239	−0.368 to 0.384	−0.576 to 0.135	−0.479 to 0.263	−0.506 to 0.230
CCL5	−0.129 to 0.579	**−0.674 to -0.0257**	−0.627 to 0.0546	−0.287 to 0.459	−0.595 to 0.105	−0.0515 to 0.629
TNF-α	−0.315 to 0.434	−0.583 to 0.124	−0.371 to 0.381	−0.488 to 0.252	−0.526 to 0.204	−0.393 to 0.360

Plasma IL-1ra had a mono-exponential decay relationship (adjusted R^2^ = 0.456) with the HCC/NAB K^trans^ ratio, and (Fig. 4c): the distribution of the residuals passed the normality test (Shapiro Wilk test, P value = 0.284). The empirical equation for the relationship between HCC/NAB Ktrans ratio (Y) and IL-1ra plasma concentration (X, pg/ml) was as follows:

$$Y=\left(52.76-10.18\right)\times e^{-0.03426X}+10.18$$

IL-1ra did not have significant relationships with NAB K^trans^ (Fig. 4b): slopes were not significantly different from zero. No other plasma immune markers could be fitted into a linear or non-linear relationship with the quantified imaging metrics.

TBI participants' data, including injury severity, GCS, age, imaging time-point post-injury, HCC K^trans^, NAB K^trans^, and HCC/NAB K^trans^ ratio, and the plasma concentrations of the different immunological markers and the healthy controls’ plasma concentrations of the different immunological markers are presented in Supplementary Table 1.

Since the causes and mechanisms of injuries of the patients in this study were known to cause extra-cranial injuries and those injuries may cause changes to the cytokine levels in the plasma, additional analysis were performed to rule out the effects of extra cranial injuries. Details available from patients’ files on extra-cranial injuries are presented in Supplementary Table 2. The analysis results presented in this section based on all participants were replicated for only the participants without extra-cranial injuries in Supplementary Data 3.

## 4 Discussion

TBI is widely accepted to initiate neuroinflammation via activation of astrocytes and microglial, in conjunction with increased BBB leakage, as evidenced by loss of pericytes and parenchymal accumulation of serum components. Indeed, any disturbance of BBB integrity will promote the exchange of inflammatory markers between nervous tissue and blood, although it is unknown to what extent peripheral markers measured in the plasma reflect CNS damage in TBI. In this study, we found significant group differences in individual plasma cytokine levels between controls and TBI patients. Among the various analytes, IL-1ra significantly correlated with BBB damage measured by DCE MRI. While the causal relationship remains unknown, present findings may present biomarkers predictive of outcome after TBI.

### 4.1 Peripheral plasma immunological marker profiles post-TBI

The plasma levels of IL-1β, IFN-γ, IL-13, and CCL2 were lower, and the levels of PDGF-BB, IL-6, and IL-8 were higher in TBI patients compared to healthy controls. The relative reduction in IL-1β post-TBI may appear counterintuitive, given the expectation of neuroinflammation post-TBI and earlier results showing elevated brain levels of IL-1β post-TBI rat models of TBI (Griffin et al., 1994; Hutchinson et al., 2007a; Newell et al., 2018; Fan et al., 1995). However, we note that those earlier results were obtained within 24 h of injury (Newell et al., 2018; Fan et al., 1995; Kamm et al., 2006). In particular, in the drop weight model of TBI, brain IL-1β levels increased within the first hour, peaked at 16 h, and resolved within 24 h post-injury without concomitant changes in plasma or liver concentrations (Kamm et al., 2006). In other studies, higher CSF levels of IFN-γ, IL-6, IL-8, and IL-1β (among other markers) were associated with unfavourable outcomes post-TBI in human patients (Nwachuku et al., 2016). Higher plasma levels of CCL2 were associated with higher injury severity and worse white matter integrity in TBI patients (Ho et al., 2020). Furthermore, higher plasma levels of IL-6 (Abboud et al., 2016), IL-1β (Di Battista et al., 2016), IL-8 (Abboud et al., 2016; Di Battista et al., 2016), CCL2 (Di Battista et al., 2016), (among other markers such as TNF-α (Rodney et al., 2018)) and IL-6 (Maier et al., 2007) were also associated with post-TBI mortality, whereas higher levels of IL-6 (Woiciechowsky et al., 2002; Aisiku et al., 2016) and IL-8 (Aisiku et al., 2016) were associated with respiratory distress in TBI patients.

We detected increased levels of PDGF-BB in plasma of TBI patients. This factor inhibited endoplasmic reticulum and autophagy stress and promoted recovery post-TBI in a mouse model of open-head TBI (Wu et al., 2022). In another rodent model, TBI interfered with PDGF signalling, which contributed to dysfunction of the BBB (Bhowmick et al., 2019). Overall, the present post-TBI plasma results showed trends that might plausibly promote both favourable and unfavourable outcomes post-TBI. This may reflect the inherent complex interaction between brain injury and inflammation, in the brain, systemic, or in the context of infection. Following a TBI, proinflammatory responses are necessary for the healing of contused brain tissues and protects against secondary infections (Bouras et al., 2022). On the other hand, an exacerbated systemic inflammatory response can induce multiple organ failures with very high mortality rate (Cole et al., 2020). In response, an anti-inflammatory response may be initiated but that, in turn, creates a risk of secondary infection, such as nosocomial infection or ventilator-related pneumonia, which are a different and independent risk factor for unfavourable outcomes (Bouras et al., 2022). The mixed results may relate to the unavoidably variable time between injury and scanning or blood sampling; we could only scan patients upon their medical stabilization, which tended to require a longer delay in patients with a more severe injury. The imaging time-point was also dependent on the scanner availability.

### 4.2 Relationship between plasma levels of IL-1ra and BBB integrity as measured by DCE-MRI

Analysis of correlates of the imaging measures of cerebral microbleeds and BBB integrity showed that only the plasma levels of IL-1ra were associated with imaging metrics of BBB integrity (HCC/NAB K^trans^). Specifically, higher plasma levels of IL-1ra were associated with a lower HCC/NAB K^trans^ ratio near the primary lesion, predicting lesser BBB dysfunction. A mono-exponential decay function gave the best description of this relationship. IL-1ra is an endogenous inhibitor of the pro-inflammatory cytokine IL-1, which has important effects both on the immune system and the CNS (Allan et al., 2005). IL-1ra is an important anti-inflammatory protein in human autoimmune diseases such as arthritis, colitis, and granulomatous pulmonary disease (Arena et al., 1998), and has attracted some interest as a target in CNS disorders with an inflammatory component (Allan et al., 2005). Higher IL-1ra levels and higher IL-1ra/IL-1β ratio in brain microdialysis samples within the first 24 h were associated with more favourable outcomes in severe human TBI patients (Rodney et al., 2018; Hutchinson et al., 2007b). As previously discussed with regards to IL-1β, while blood and brain levels of immune markers do not necessarily track each other or correlate (Zetterberg and Blennow, 2016; Morganti-Kossmann et al., 2002; Thelin et al., 2017; McDonald et al., 2016), peripheral administration of recombinant human IL-1ra resulted in increased IL-1ra levels in human microdialysis CSF samples (Helmy et al., 2014). However, the same clinical trial showed a generally poor agreement between immune marker profiles in blood and the brain extracellular fluid, both simultaneously or with a temporal delay (Lassarén et al., 2021). While it might be expected that systemic inflammatory changes affect would affect cytokine profiles in the brain, there are reports to the contrary: in systemic clinical infection with increased blood levels of several cytokines, brain extracellular fluid samples showed decreased levels IL-1ra, G-CSF, PDGF-BB, CCL4, and CCL5 (Lassarén et al., 2021). While various immune markers had increased or decrease plasma levels in the present TBI group, these changes were not consistently associated with changes in BBB integrity as measured by DCE-MRI. In an exception to this general rule, plasma IL-1ra had a positively correlated relationship with BBB integrity (higher IL-1ra levels was associated with more intact BBB), despite the absence of significant elevation in plasma IL-1ra levels in the TBI group.

The distributions of plasma levels of IL-1ra among TBI participants and healthy controls provide a clue towards resolving this conundrum. TBI participants and healthy controls had similar median IL-1ra levels, with comparable extreme outliers with very high IL-1ra levels, which argues against any causal relationship between individual levels and the brain injury. We suppose that the observed relationship between BBB integrity and individual IL-1ra levels might rather reflect trait differences in the regulation of the BBB, or the multiplicity of cellular sources of IL-1ra (Volarevic et al., 2010), rather than a response to the injury *per se*. A clinical trial on the effects of IL-1ra administration post-TBI was inconclusive about its possible benefits, and did not test for effects on BBB integrity (Helmy et al., 2014). The present exploratory findings nonetheless suggest an association whereby individual plasma levels of IL-1ra covary with the extent of BBB impairment after TBI.

### 4.3 Limitations

We have above alluded to the variable times post-injury across TBI patients. Although imaging and blood collection took place on the same day, phasic temporal changes in the BBB integrity and immune marker profiles may have obscured their association.

## 5 Conclusion

In this study, we replicate general findings of altered plasma cytokine levels in the aftermath of TBI in a series of 30 patients. While IL-1ra levels were seemingly unaffected by TBI, there emerged a significant inverse correlation between individual IL-1ra plasma levels and BBB integrity as measured by DCE-MRI. The results may reflect the ability of plasma IL-1ra to cross the BBB, thus drawing attention to the potential of IL-1ra as a target for interventions against the progression of TBI.

## Declaration of competing interest

The authors have no conflict of interest to declare.

## Data Availability

Data will be made available on request.

## Appendix A Supplementary data

Multimedia component 1 **Supplementary data 3**: replicated statistical results similar to that presented in *Relationship between plasma levels of immunological markers and imaging outcomes* section of the Results, Table 1 and Fig. 4 but among Traumatic Brain Injury participants without any extra-cranial injury. Multimedia component 1

Multimedia component 2 **Supplementary data 1**: full data table of all the quantified variables used in the correlation analysis. The lowest standard concentration used to construct the standard curve in immune biomarker quantification was presented. Some values below this lowest concentration standard were extrapolated from the standard curve. The values below the detectable range was considered to be zero for the purpose of analysis. Multimedia component 2

Multimedia component 3 **Supplementary data 2**: details of extra-cranial injuries in among the Traumatic Brain Injured patients. N = None. Multimedia component 3
