# Supplementary material for: Anti-inflammatory interleukin 1 receptor antagonist concentration in plasma correlates with blood-brain barrier integrity in the primary lesion area in traumatic brain injury patients
Source: Brain Behav Immun Health. 2023 Jun 22;31:100653. doi: 10.1016/j.bbih.2023.100653 (PMC10320227; doi:10.1016/j.bbih.2023.100653)

|  | severity | GCS | Age | HCC Ktrans | NAB ktrans | HCC/NAB ratio |
| --- | --- | --- | --- | --- | --- | --- |
| severity |  | **-0.866 to -0.373** | -0.666 to 0.121 | -0.206 to 0.615 | -0.515 to 0.342 | **0.0803 to 0.764** |
| GCS | -0.866 to -0.373 |  | -0.0365 to 0.711 | -0.499 to 0.361 | -0.168 to 0.639 | **-0.687 to 0.0831** |
| Age | -0.666 to 0.121 | -0.0365 to 0.711 |  | -0.366 to 0.494 | -0.293 to 0.554 | -0.574 to 0.266 |
| HCC Ktrans | **-0.206 to 0.615** | -0.499 to 0.361 | -0.366 to 0.494 |  | -0.721 to 0.0154 | 0.565 to 0.916 |
| NAB ktrans | -0.515 to 0.342 | -0.168 to 0.639 | -0.293 to 0.554 | -0.721 to 0.0154 |  | -0.780 to -0.118 |
| HCC/NAB ratio | **0.0803 to 0.764** | -0.687 to 0.0831 | -0.574 to 0.266 | 0.565 to 0.916 | -0.780 to -0.118 |  |
| IL-1β | -0.588 to 0.246 | -0.386 to 0.477 | -0.0315 to 0.714 | -0.722 to 0.0149 | -0.0793 to 0.689 | **-0.736 to -0.0156** |
| IL-1ra | -0.711 to 0.0362 | -0.321 to 0.532 | -0.254 to 0.583 | -0.683 to 0.0913 | -0.509 to 0.350 | **-0.770 to -0.0952** |
| IL-4 | -0.278 to 0.565 | -0.661 to 0.130 | -0.107 to 0.674 | -0.271 to 0.570 | -0.663 to 0.127 | -0.114 to 0.670 |
| IL-6 | -0.282 to 0.562 | **-0.818 to -0.221** | **-0.738 to -0.0209** | -0.598 to 0.232 | -0.530 to 0.324 | -0.397 to 0.467 |
| IL-7 | -0.188 to 0.626 | -0.661 to 0.131 | -0.417 to 0.448 | -0.333 to 0.522 | -0.613 to 0.210 | -0.0769 to 0.690 |
| IL-8 | -0.438 to 0.427 | -0.636 to 0.172 | -0.402 to 0.462 | -0.650 to 0.150 | -0.507 to 0.351 | -0.567 to 0.276 |
| IL-9 | -0.372 to 0.489 | -0.629 to 0.184 | -0.259 to 0.579 | -0.488 to 0.373 | -0.561 to 0.283 | -0.388 to 0.475 |
| IL-13 | -0.572 to 0.268 | 0.0649 to 0.758 | -0.113 to 0.671 | -0.727 to 0.00253 | 0.0143 to 0.735 | -0.737 to -0.0187 |
| IL-17 | -0.484 to 0.378 | -0.446 to 0.419 | **0.0711 to 0.760** | -0.592 to 0.240 | -0.501 to 0.358 | -0.452 to 0.412 |
| Eotaxin | -0.261 to 0.577 | -0.685 to 0.0877 | -0.264 to 0.575 | 0.0228 to 0.739 | -0.818 to -0.220 | **0.0912 to 0.769** |
| bFGF | -0.534 to 0.319 | -0.480 to 0.382 | -0.248 to 0.587 | -0.677 to 0.102 | -0.530 to 0.324 | -0.501 to 0.358 |
| G-CSF | -0.443 to 0.421 | -0.533 to 0.320 | -0.497 to 0.363 | -0.692 to 0.0742 | -0.610 to 0.213 | -0.525 to 0.329 |
| IFN-γ | -0.641 to 0.164 | -0.174 to 0.635 | -0.0751 to 0.691 | **-0.817 to -0.218** | **0.00920 to 0.733** | **-0.850 to -0.319** |
| CXCL10 | -0.437 to 0.428 | -0.499 to 0.361 | -0.623 to 0.193 | -0.277 to 0.566 | -0.683 to 0.0904 | -0.297 to 0.551 |
| CCL2 | 0.0517 to 0.752 | -0.669 to 0.116 | -0.190 to 0.625 | -0.447 to 0.418 | -0.585 to 0.251 | -0.348 to 0.510 |
| CCL3 | -0.463 to 0.400 | -0.536 to 0.316 | -0.557 to 0.289 | -0.563 to 0.280 | -0.691 to 0.0764 | -0.423 to 0.441 |
| PDGF-BB | -0.251 to 0.584 | **-0.736 to -0.0166** | -0.525 to 0.329 | -0.572 to 0.269 | -0.622 to 0.195 | -0.317 to 0.535 |
| CCL4 | -0.435 to 0.430 | -0.541 to 0.310 | -0.274 to 0.568 | -0.721 to 0.0158 | -0.446 to 0.419 | -0.609 to 0.216 |
| CCL5 | -0.156 to 0.646 | **-0.747 to -0.0407** | -0.455 to 0.410 | -0.436 to 0.429 | -0.675 to 0.106 | -0.241 to 0.591 |
| TNF-α | -0.521 to 0.335 | -0.564 to 0.280 | -0.209 to 0.613 | -0.599 to 0.231 | -0.528 to 0.326 | -0.432 to 0.433 |

**Supplementary Table 3**: 95% confidence intervals of Spearman correlation coefficient of the quantified plasma immunological markers and initial injury severity, Glasgow Coma Scale, Age, and the transfer constant (K^trans^) from the Haemorrhagic Contusion Core (HCC), Normal Appearing Brain (NAB), and the ratio of K^trans^ of the HCC and NAB, among participants without extra-cranial injuries,

Among participants without other recorded extra-cranial injuries, plasma IL-1ra level followed a mono-exponential decay relationship (adjusted R^2^ = 0.177) with HCC K^trans^, with the distribution of the residuals passing the normality test (Shapiro Wilk test, P value = 0.2897). The empirical equation for the relationship between HCC K^trans^ (Y, min^-1^) and IL-1ra plasma concentration (X, pg/ml) was as follows:


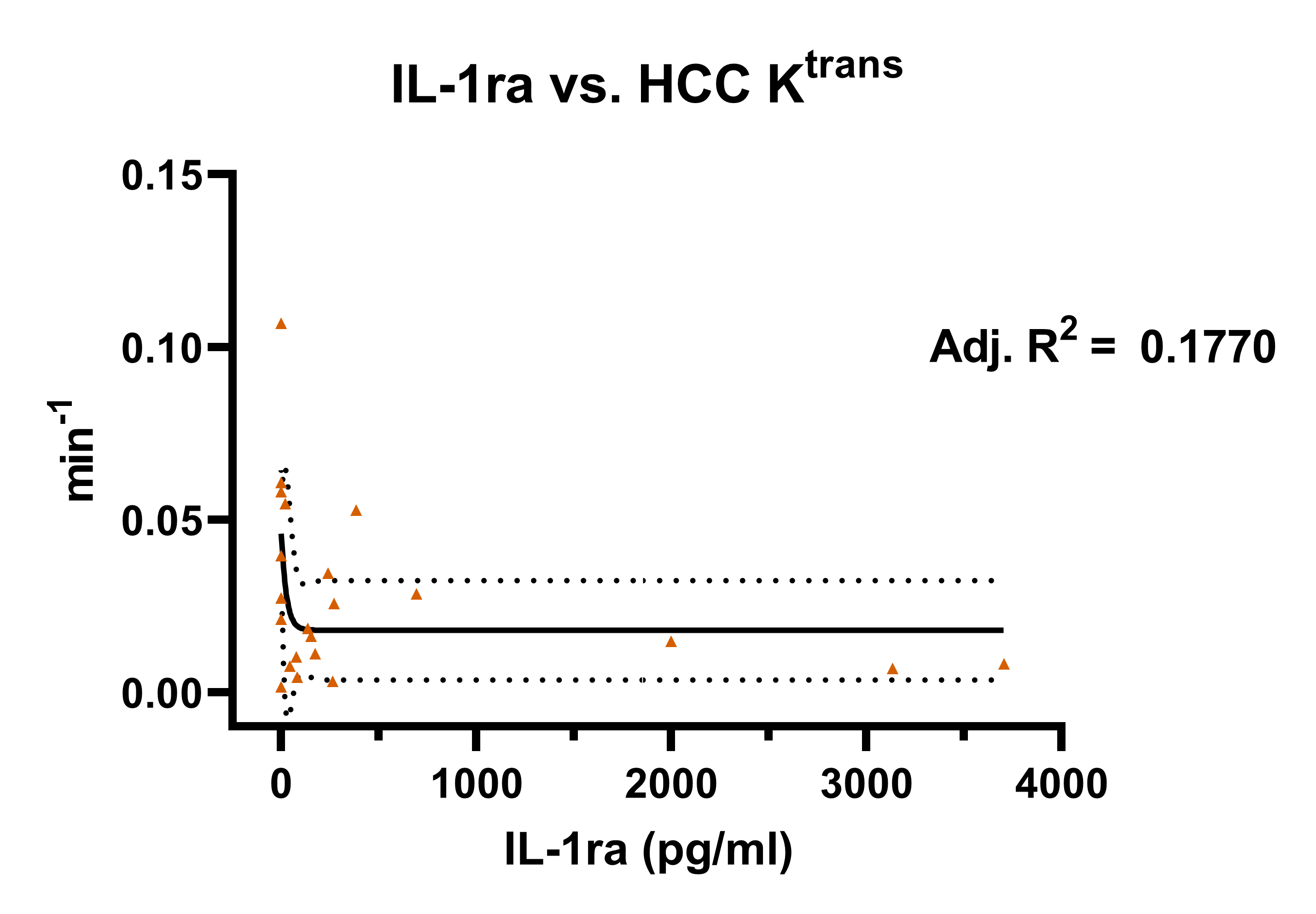

$$Y=\left( 0.04598-0.01795 \right)\times e^{-0.082 X}+0.01795$$

Among TBI patients without extra-cranial injuries, plasma IL-1ra had a mono-exponential decay relationship (adjusted R^2^ = 0.41) with the HCC/NAB K^trans^ ratio: the distribution of the residuals passed the normality test (Shapiro Wilk test, P value = 0.6401). The empirical equation for the relationship between HCC/NAB Ktrans ratio (Y) and IL-1ra plasma concentration (X, pg/ml) was as follows:

$$Y=\left( 52.71-10.62 \right)\times e^{-0.03311 X}+10.68$$

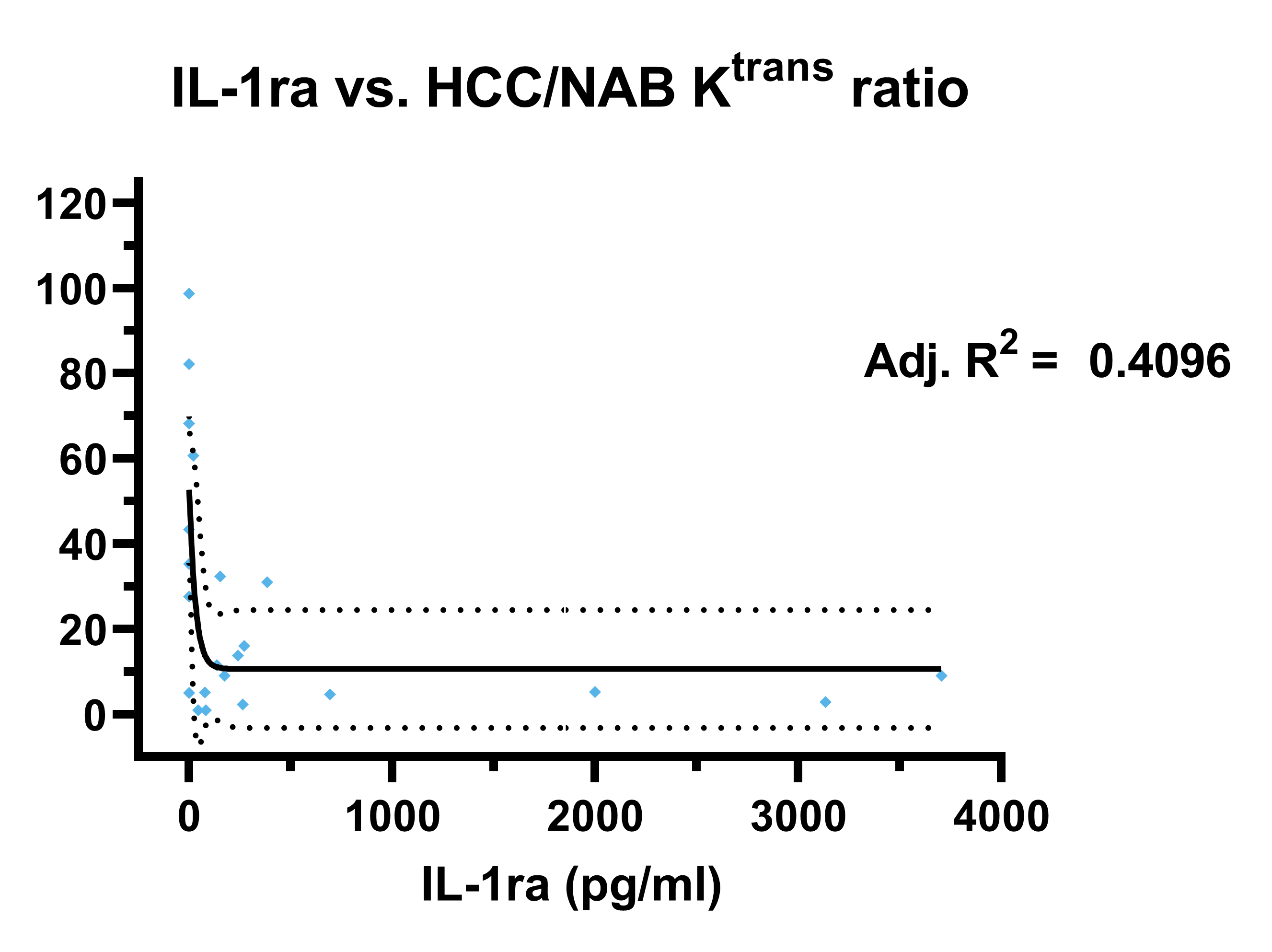

Supplement: Multimedia component 1 — Supplementary data 3: replicated statistical results similar to that presented in Relationship between plasma levels of immunological markers and imaging outcomes section of the Results, Table 1 and Fig. 4 but among Traumatic Brain Injury participants without any extra-cranial injury. [file mmc1.docx]
